# The Metallodrug BOLD-100 Is a Potent Inhibitor of SARS-CoV-2 Replication and Has Broad-Acting Antiviral Activity

**DOI:** 10.3390/biom13071095

**Published:** 2023-07-08

**Authors:** Daniel S. Labach, Hinissan P. Kohio, Edwin A. Tse, Ermela Paparisto, Nicole J. Friesen, Jim Pankovich, Mark Bazett, Stephen D. Barr

**Affiliations:** 1 Department of Microbiology and Immunology, Schulich School of Medicine and Dentistry, Western University, Dental Sciences Building Room 3007, London, ON N6A 5C1, Canada; dlabach@uwo.ca (D.S.L.);; 2 Bold Therapeutics Inc., 422 Richards St, Suite 170, Vancouver, BC V6N 2Z4, Canadamb@bold-therapeutics.com (M.B.)

**Keywords:** BOLD-100, ruthenium-based drug, SARS-CoV-2, COVID-19, HIV-1, adenovirus, antiviral

## Abstract

The COVID-19 pandemic has highlighted an urgent need to discover and test new drugs to treat patients. Metal-based drugs are known to interact with DNA and/or a variety of proteins such as enzymes and transcription factors, some of which have been shown to exhibit anticancer and antimicrobial effects. BOLD-100 (sodium *trans*-[tetrachlorobis(1*H*-indazole)ruthenate(III)]dihydrate) is a novel ruthenium-based drug currently being evaluated in a Phase 1b/2a clinical trial for the treatment of advanced gastrointestinal cancer. Given that metal-based drugs are known to exhibit antimicrobial activities, we asked if BOLD-100 exhibits antiviral activity towards SARS-CoV-2. We demonstrated that BOLD-100 potently inhibits SARS-CoV-2 replication and cytopathic effects in vitro. An RNA sequencing analysis showed that BOLD-100 inhibits virus-induced transcriptional changes in infected cells. In addition, we showed that the antiviral activity of BOLD-100 is not specific for SARS-CoV-2, but also inhibits the replication of the evolutionarily divergent viruses Human Immunodeficiency Virus type 1 and Human Adenovirus type 5. This study identifies BOLD-100 as a potentially novel broad-acting antiviral drug.

## 1. Introduction

Vaccination is widely recognized to be the most important component of severe COVID-19 prevention. Several mAb-based therapies have received Emergency Use Authorization, such as bamlanivimab/etesevimab, casirivimab/imdevimab, sotrovimab, and bebtelovimab. These mAbs recognize and bind a specific epitope present on the receptor-binding domain (RBD) of the SARS-CoV-2 S protein, thereby precluding interaction with its ACE2 receptor on the cells and neutralizing the virus. Generally, clinical studies have shown that mAb administration reduces the risk of COVID-19 patients requiring hospitalization [1]. However, the emergence and ubiquitous spread of the SARS-CoV-2 omicron variant has rendered established mAbs less effective at neutralizing infection, underscoring the challenge in developing therapies that target the specific viral epitopes that are subject to mutation [2].

As vaccines are prophylactic by nature, therapeutics fill an important role in the treatment of infected patients at risk for progression to severe disease. Currently, Veklury (Remdesivir), Paxlovid (Nirmatrelvir/Ritonavir), Actemra (Tocilizumab), and Olumiant (Baricitinib) are approved by the United States Food and Drug Administration (F.D.A.) for the treatment of COVID-19. Remdesivir is an intravenously administered agent with broad-spectrum antiviral activity and is the only antiviral drug that is approved by the F.D.A. for the treatment of COVID-19. Upon its entry into cells, it is metabolized into an ATP nucleoside analogue with a higher affinity for the viral RdRp than ATP and, as a result, interferes with viral RNA synthesis [3]. While remdesivir is effective at inhibiting SARS-CoV-2 replication in cell culture and has been shown to reduce clinical disease in rhesus macaques, its efficacy in real-world settings has remained contentious [4,5,6]. The World Health Organization Solidarity trial, which evaluated COVID-19 drug candidates in over 400 hospitals worldwide, found that remdesivir had no effect on mortality or hospitalization duration in ventilated patients, and exhibited only a small effect against death or the initiation of ventilation in hospitalized patients [7].

Nirmatrelvir is an inhibitor of SARS-CoV-2 Mpro that covalently binds a cysteine residue responsible for the viral protease’s catalytic activity [8]. Mpro is essential for viral replication, cleaving most of the non-structural proteins during the replication cycle, and is highly sequence-specific, limiting the collateral inhibition of the host’s proteases via nirmatrelvir. In this drug mixture, the metabolic degradation of nirmatrelvir is slowed by ritonavir, which inhibits the metabolic enzyme CYP3A4 [9,10]. In a phase 2/3 clinical trial, Paxlovid treatment resulted in an 89% reduction over a placebo with regard to progression to severe COVID-19 [11]. Recently, case reports have emerged that detail a “rebound” of COVID-19 after the completion of Paxlovid treatment.

Lagevrio (Molnupiravir) is the oral prodrug of the ribonucleoside analog beta-D-N4-hydroxycytidine (NHC). Once formed, NHC converts into the active 5**′**-triphosphate form, which serves as a competitive substrate for virally encoded RNA-dependent RNA polymerase (RdRp), resulting in an accumulation of errors in the nascent viral RNA [12,13,14,15,16]. In a phase 2a clinical trial, Lagevrio treatment for patients with COVID-19 reduced the infectious virus replication and interrupted the progression of COVID-19 during the early stages of the disease [17]. Lagevrio has received Emergency Use Authorization for the treatment of COVID-19.

Actemra is a monoclonal antibody that reduces inflammation by blocking the interleukin-6 receptor and has been shown to reduce the likelihood of needing mechanical ventilation or death [18]. Baricitinib is a Janus kinase 1/2 inhibitor with known anti-inflammatory and anti-viral properties and is associated with a shorter duration of hospitalization and reduced mortality [19].

Since the emergence of SARS-CoV-2 in 2019, a great deal of effort has been focused on drug repurposing strategies to treat COVID-19 quickly and safely. Antiviral effects have previously been reported for many types of metal-based drugs, highlighting the potential for these metal-based drugs in treating COVID-19 (reviewed in [20,21,22,23]). Recently, Gil-Moles and colleagues (2021) used metallodrug profiling against SARS-CoV-2 target proteins to identify potential antiviral inhibitors and showed that some of these drugs are potent inhibitors of critical SARS-CoV-2 targets, such as the SARS-CoV-2 spike protein/host ACE2 receptor interaction and the SARS-CoV-2 papain-like protease PL^pro^ [24].

Ruthenium-based drugs came to scientific attention in the 1980s as compounds that were observed to preferentially localize in tumor tissue and exhibit a lower toxicity than traditional platinum-based chemotherapeutics [25,26,27]. Depending on the compound, its pharmacological activity may be ascribed to the metal center and/or its ligands. While the ruthenate anion may itself interact with cellular targets, it may also simply act as a scaffold to carry bioactive ligands to a target site, where they are released and exert their effects [28,29]. Metal-based drugs, including ruthenium-based compounds, act via a multitude of mechanisms, mainly involving interactions with DNA or various proteins such as enzymes and transcription factors [29]. Notably, ruthenium-based compounds have recently been shown to exhibit potent antiviral activity towards SARS-CoV-2 by targeting different SARS-CoV-2 proteins, such as papain-like protease PL^pro^ and M^pro^ [24,30,31]. Their antiviral activity against other viruses, such as Chikungunya virus, also highlights the potential of ruthenium-based compounds as broad-acting antivirals [32].

BOLD-100 is a clinical-stage ruthenium-based compound that is composed of sodium *trans*-[tetrachlorobis(1*H*-indazole)ruthenate(III)]dihydrate [33]. Its predecessor molecules include KP1339 (NKP-1339/IT-139), which has a different synthesis pathway, and KP1019, which has an indazolium counterion instead of sodium. BOLD-100 is being developed as an anticancer agent that has completed Phase 1 monotherapy studies and is currently being tested in a Phase 1b/2 clinical trial in combination with chemotherapy for advanced gastrointestinal cancer (NCT04421820) [26]. Mechanistically, BOLD-100 has a complex, multimodal mechanism of action that has not been fully characterized. In cancerous cells, BOLD-100 or its predecessor molecules have been shown to induce the unfolded protein response due to the modulation of endoplasmic reticulum chaperone 78 kDA glucose regulated protein (GRP78), induce reactive oxygen species generation, induce a DNA damage response, interact and be impacted by ribosomal biogenesis, and alter cellular metabolism [33,34,35,36].

Prior to the COVID-19 pandemic, BOLD-100 had never been tested as an antiviral. Accordingly, the existing body of literature focuses almost exclusively on the drug’s anticancer properties. Although not fully characterized, multiple components of BOLD-100′s mechanism of action have the potential to cause viral inhibition. Additionally, clinical trials in oncology patients have suggested that BOLD-100 is well tolerated with limited severe adverse events, even at high dose levels [26]. The need for novel therapeutics for the treatment of COVID-19 and other viral pathogens warrants expanded research into novel therapeutic approaches. Here, we investigated the potential of BOLD-100 as an antiviral inhibitor and demonstrated that it not only potently inhibits SARS-CoV-2 replication, but the replication of other evolutionarily diverse viruses, HIV-1 and adenovirus.

## 2. Materials and Methods

### 2.1. Cell Culture

Vero E6 cells (for SARS-CoV-2 infection) and A549 cells (for HAdV-C5 infection) were purchased from ATCC. HEK 293T-ACE2 cells (for SARS-CoV-2 infection) were obtained from BEI Resources, NIAID, NIH. HeLa-TZM-bl cells and HOS CD4+ CXCR4+ cells (for HIV-1 infection) were obtained from the NIH HIV Reagent Program, Division of AIDS, NIAID, NIH. All cell lines were maintained in standard growth medium (Dulbecco’s Modified Eagle’s Medium (DMEM)), supplemented with 10% heat-inactivated Fetal Bovine Serum (FBS), 100 U/mL of Penicillin, and 100 µg/mL of Streptomycin at 37 °C with 5% carbon dioxide (CO_2_). For the in vitro experiments involving virus infections, the viruses were diluted in DMEM supplemented with 2% FBS, 100 U/mL of penicillin, and 100 µg/mL streptomycin.

### 2.2. Viruses

The following replication-competent SARS-CoV-2 isolates were acquired from BEI Resources, NIAID, NIH: 2019/nCoV/USA-WA-I/2020, hCoV-19/England/204820464/2020, hCoV-19/South Africa/KRISP-EC-K005321/2020, and hCoV-19/USA/PHC658/2021. The following reagent was obtained through the NIH HIV Reagent Program, Division of AIDS, NIAID, NIH (Bethesda, MD, USA): Human Immunodeficiency Virus Type 1 AD.MDR01, ARP-11700, contributed by Dr. Martin Markowitz and Dr. Hiroshi Mohri. HAdV-C5 strain Adenoid 75 was purchased from ATCC.

### 2.3. Virus Propagation

SARS-CoV-2 was propagated and provided by the ImPaKT facility at Western University (London, ON, Canada). Briefly, the Vero E6 cells were grown to 70% confluency in T-150 cell culture flasks. Infectious SARS-CoV-2 (2 × 10^5^ PFU) was diluted in 5 mL of serum-free DMEM. The existing cell culture media were removed from the flask and SARS-CoV-2-containing media were added. The flask was incubated for 1 h at 37 °C and 5% CO_2_ for 1 h. The virus inoculum was then removed and 30 mL of DMEM + 2% FBS was added. The flask was incubated at 37 °C and 5% CO_2_ for 72 h, after which, the virus-containing supernatant was removed and centrifuged at 500× *g* for 10 min to pellet the cellular debris. The supernatant was removed and aliquoted into cryovials for storage at −80 °C. The virus titer was determined by a 50% tissue culture infectious dose (TCID50) assay on the Vero E6 cells [37]. Specifically, 1.5 × 10^4^ Vero E6 cells were seeded in 96-well plates 24 h prior to infection. On the day of infection, the cell culture medium was removed and the cells were washed 1x with PBS. Ten-fold serial dilutions of stock SARS-CoV-2 were prepared in DMEM + 2% FBS. The cells were infected in triplicate with 90 µL of either undiluted virus, one of the serial dilutions of virus, or media only as a negative control. The cells were incubated for 72 h at 37 °C with 5% CO_2_ and visually evaluated for cytopathogenicity to determine infection. TCID50/mL was calculated using the Reed–Muench method.

HIV-1 AD.MDR01 was propagated in U87.R5 cells. Briefly, the U87.R5 cells were seeded in T-150 cell culture flasks. Once the cells reached 80% confluency, the cell culture medium was removed. A 1 mL aliquot of stock virus was diluted in 30 mL of DMEM + 2% FBS and added to the flask. The cells were incubated at 37 °C with 5% CO_2_ for 7 days. The virus-containing supernatant was harvested from the flask and centrifuged at 500× *g* for 10 min to pellet the cell debris. The resulting supernatant was aliquoted into cryovials and stored at -80 °C. The virus titer was determined by a TCID50 assay on TZM-bl cells. Specifically, 1.5 × 10^4^ TZM-bl cells were seeded in 96-well plates 24 h prior to infection. On the day of infection, the cell culture medium was removed. Ten-fold serial dilutions of propagated HIV-1 AD.MDR01 were prepared in DMEM + 2% FBS. The cells were infected in triplicate with 90 µL of either undiluted virus, one of the serial dilutions of virus, or media only as a negative control. The cells were incubated for 48 h at 37 °C with 5% CO_2_ and the infection in each well was determined using the Galacto-Star™ β-Galactosidase Reporter Gene Assay System (ThermoFisher, Waltham, MA, USA). The TCID50/mL was calculated using the Reed–Muench method.

HAdV-C5 strain Adenoid 75 was propagated in HEK 293T cells. Briefly, the HEK 293T cells were grown to 60% confluency in T-75 cell culture flasks. The cell culture media was removed and the cells were washed 1× with PBS before the addition of HAdV-C5 diluted in 4 mL of DMEM + 2% FBS (MOI = 1). The flasks were incubated at 37 °C with 5% CO_2_ with periodic rocking for 3 h, after which, 10 mL of DMEM + 10% FBS was added to each flask. The flasks were incubated at 37 °C with 5% CO_2_ for 72 h, after which, the contents were removed and centrifuged at 500× *g* for 10 min to pellet the cells. The supernatant was discarded and the cells were resuspended in sterile PBS (1 mL per T-75 flask). The resuspended cells were subjected to 3 freeze–thaw cycles to liberate the virus, after which, the virus-containing solution was centrifuged at 500× *g* for 10 min to pellet the cellular debris. The supernatant was aliquoted into cryovials and stored at −80 °C. The virus titer was determined by a plaque assay on A549 cells.

### 2.4. BOLD-100 Preparation

BOLD-100 (sodium *trans*-[tetrachlorobis(IH-indazole)ruthenate(III)]dihydrate with cesium as an intermediate salt form) drug substance powder was provided by Bold Therapeutics Inc. For the in vitro experiments, the BOLD-100 powder was dissolved in dimethylsulfoxide (DMSO) to generate a stock solution and was further diluted in cell culture media for working solutions (final DMSO concentration was kept below 0.1%).

### 2.5. SARS-CoV-2 Infections

Twenty-four hours prior to infection, 1.5 × 10^4^ Vero E6 or 293T-ACE2 cells were seeded in 96-well plates. SARS-CoV-2 isolates were diluted in cell culture media and added to the cells for 1 h at 37 °C with 5% CO_2_ for the adsorption of viral particles. The virus inoculum was then removed and fresh media supplemented with BOLD-100 were added. Either 24 or 48 h post-infection, the cell lysates and/or virus-containing supernatants were harvested for a downstream analysis. Specifically, the plates containing cells were centrifuged at 500× *g* for 10 min and the virus-containing supernatants were removed. The cell lysates were prepared by resuspending the remaining cells in either radioimmunoprecipitation buffer (50 mM Tris-HCl (pH 7.4), 150 mM NaCl, 0.5% sodium deoxycholate, and 0.5% sodium dodecyl sulfate (SDS)) for Western blot or RNA lysis buffer (PureLink RNA Mini Kit #12183020; ThermoFisher, Waltham, MA, USA) for RNA extraction.

### 2.6. HIV-1 Infections

The TZM-bl cells were seeded in 96-well plates (2 × 10^4^ cells/well) 24 h prior to infection. TZM-bl cells are a HeLa derivative that express the HIV-1 receptor CD4 and the CCR5 co-receptor, in addition to β-galactosidase and firefly luciferase under the control of a Tat-responsive HIV-1 long terminal repeat promoter (NIH HIV Reagent Program, Division of AIDS, NIAID, NIH (Bethesda, MD, USA)), #ARP5011) [38,39,40]. This approach enabled the quantification of infectious virus in the cell culture by measuring the β-galactosidase activity. On the day of infection, the cells were infected with HIV-1 AD.MDR01 at MOI 0.05 and treated with or without varying concentrations of BOLD-100. At 48 h post-infection, the β-galactosidase activity was measured in the cell lysates using the Galacto-Star™ β-Galactosidase Reporter Gene Assay System (ThermoFisher, Waltham, MA, USA).

### 2.7. HAdV-C5 Infections and Plaque Assay

The A549 cells were seeded in 12-well plates (2 × 10^5^ cells/well) 24 h prior to infection. The cells were washed 1x with PBS and infected with the HAdV-C5 strain Adenoid 75 (ATCC VR-5) (MOI = 1) for 1 h. The virus-containing media were removed and replaced by fresh media supplemented with BOLD-100. At 48 h post-infection, the cells were collected from each well and subjected to 3 freeze–thaw cycles to liberate the virus, after which, the virus-containing solution was centrifuged at 500× *g* for 10 min to pellet the cellular debris. The viral titers were determined by a plaque assay. Briefly, the confluent monolayers of the A549 cells were infected with 400 µL of virus-containing media for one hour, with the plates being rocked every 10 min. The virus inoculum was then removed and 3 mL of Avicel overlay was added (1:1 mixture of 2.4% Avicel with plaque media (2X MEM, 4% FBS, 200 U/mL penicillin, 200 µg/mL streptomycin)). The plates were incubated for 5 days at 37 °C and 5% CO_2_, and subsequently fixed with 10% formaldehyde and stained with crystal violet to visualize the plaques.

### 2.8. Cell Viability Assays

For a determination of the cell viability using a CellTiter-Glo viability assay (Promega, Madison, WI, USA), 1.5 × 10^4^ Vero E6 cells were seeded in 96-well plates the day prior to the BOLD-100 treatment and infected with SARS-CoV-2. The cells were infected with SARS-CoV-2 (MOI = 0.01) or mock-infected with diluent alone for a 1 h adsorption period, after which, the inoculum was removed and fresh media supplemented with BOLD-100 were added. Seventy-two hours post-infection, the relative adenosine triphosphate (ATP) levels were measured in each well as a measure of the cell viability, according to the manufacturer’s instructions. Random brightfield images were obtained at 20× magnification using an EVOS M7000 imaging system (ThermoFisher Scientific, Waltham, MA, USA). For a determination of the cell viability using a Cell Counting Kit-8 (CCK8) assay (Sigma), the cells were seeded in 96-well plates (1.5 × 10^4^ cells/well) and incubated at 37 °C and 5% CO_2_ for 24 h. Subsequently, the cells were treated with varying concentrations of BOLD-100. Forty-eight hours post-treatment, the relative cell viability was determined using a CCK8 assay (Sigma, Oakville, ON, Canada) according to the manufacturer’s instructions. The absorbance values at 450 nm in the wells containing BOLD-100-treated cells were corrected for the background absorbance contributed by the cell culture medium and normalized to the absorbance values in the wells containing mock-treated cells to determine the relative cell viability. The 50% cytotoxic concentration (CC_50_) was calculated via nonlinear regression (Sigmoidal, 4PL, X is concentration).

### 2.9. Antibodies and Western Blotting

Rabbit anti-GAPDH (1 mg/mL) was purchased from Sigma (Oakville, ON, Canada) and diluted 1:20,000 in Phosphate Buffered Saline-Tween (1.37 M NaCl, 27 mM KCl, 100 mM Na_2_HPO_4_, and 18 mM KH_2_PO_4_, 1% Tween) (PBS-T) prior to use. Mouse anti-SARS/SARS-CoV-2 Coronavirus Nucleocapsid Monoclonal Antibody (E16C) (0.1 mg/mL) was purchased from ThermoFisher Scientific (Waltham, MA, USA) and diluted 1:2000 in PBS-T prior to use. IRdye® 800 CW goat anti-rabbit IgG (1 mg/mL) (1:20,000 dilution in PBS-T) and IRdye® 680RD goat anti-mouse IgG (1 mg/mL) (1:20,000 dilution in PBS-T) were purchased from LI-COR BioScience (Lincoln, NE, USA).

### 2.10. Western Blotting

Cell lysates were mixed with 4x protein loading dye (40% glycerol, 240 mM Tris/HCl pH 6.8, 8% SDS, 0.04% bromophenol blue, and 5% betamercaptoethanol) in a 1:3 dye to sample ratio and heated at 95 °C for 20 min to denature the proteins. Twenty μL of each cell lysate sample and 0.7 μL of BLUeye Protein Ladder (FroggaBio, Concord, ON, Canada) were loaded onto a 10% SDS-PAGE gel. The gel was submerged in 1x Protein Running Buffer (12 mM Tris base, 96 mM glycine, and 2 mM SDS in ddH_2_O) and run at 90 V for 2 h. The protein was transferred onto a 0.2 µm Low Fluorescence Amersham Hybond Polyvinylidene difluoride (PVDF) membrane (GE Healthcare Life Sciences, Piscataway, NJ, USA) via a semi-dry transfer with Transfer Buffer (8mM Tris Base, 190 mM glycine, and 20% methanol in ddH_2_O) for 90 min at 20 V. The PVDF membrane was incubated in 10 mL of a 50:50 solution of LI-COR Odyssey®️ Blocking Buffer and Phosphate Buffered Saline (PBS) (1.37 M NaCl, 27 mM KCl, 100 mM Na_2_HPO_4_, and 18 mM KH_2_PO_4_) for 60 min. The membrane was incubated overnight at 4 °C with the primary antibodies rabbit anti-GAPDH (1:20,000 dilution) and mouse anti-SARS-CoV-2 N (1:2000 dilution). The membrane was washed thrice with PBS-T for 10 min and probed with the secondary antibodies IRdye® 800 CW goat anti-rabbit IgG (1:20,000 dilution) and IRdye® 680RD goat anti-mouse IgG (1:20,000 dilution). The membrane was washed thrice for 10 min in PBS-T, scanned using the LI-COR Odyssey machine (an infrared light scanner), and visualized with the Odyssey v3.0 software (LI-COR BioScience, Lincoln, NE, USA). A densitometric analysis was performed using ImageJ 1.53 g 64-bit software (NIH, USA).

### 2.11. RNA Extraction and Quantitative RT-PCR

The total RNA from the cell lysates or supernatants was extracted using the PureLink RNA mini kit (Ambion, Life Technologies, ThermoFisher, Waltham, MA, USA) according to the manufacturer’s instructions, except in the case of the HIV-1 antiviral assay, where the viral RNA was extracted from the infected cell supernatants using the MagMax-96 Viral RNA Isolation kit (Applied Biosystems, ThermoFisher, Waltham, MA, USA), according to the manufacturer’s instructions. Each quantitative RT-PCR (RT-qPCR) reaction consisted of 1–10 µL of sample RNA, 5 µL of TaqMan™ Fast Virus 1-Step Master Mix, 1 µL of gene-specific TaqMan™ Gene Expression Assays (20X), and ribonuclease-free water to bring the total reaction volume to 20 µL. The relative levels of RNA were measured via RT-qPCRs using the QuantStudio5 qPCR machine (Applied Biosystems, ThermoFisher, Waltham, MA, USA), using the following cycling conditions: 5 min at 50 °C, 20 sec at 95 °C, and 40 cycles of 3 sec at 95 °C and 30 sec at 60 °C. TaqMan™ Fast Virus 1-Step Master Mix permits RNA quantification via an RT-qPCR without a prior synthesis of the complementary DNA in a separate reaction. The following TaqMan™ Gene Expression Assays were used: SARS-CoV-2 N gene (Vi07918637_s1), HIV-1 LTR (Vi03453409_s1), and GAPDH (Hs02786624_g1). The relative fold changes were calculated using the 2^−ΔΔCt^ method.

### 2.12. RNA Sequencing

The total RNA samples were quantified using the NanoDrop (ThermoFisher Scientific, Waltham, MA, USA) and their quality was assessed using the Agilent 2100 Bioanalyzer (Agilent Technologies Inc., Palo Alto, CA, USA) and RNA 6000 Nano kit (Caliper Life Sciences, CA, USA). They were then processed using the Vazyme VAHTS Total RNA-seq (H/M/R) Library Prep Kit for Illumina (Vazyme, Nanjing, China), which includes ribosomal RNA (rRNA) reduction. Briefly, the samples were rRNA depleted and fragmented and the cDNA was synthesized, indexed, cleaned-up, and amplified via PCRs. The libraries were then equimolar pooled into one library and their size distribution was assessed on an Agilent High Sensitivity DNA Bioanalyzer chip and quantified using the Qubit 2.0 Fluorimeter (ThermoFisher Scientific, Waltham, MA, USA). The library was sequenced on an Illumina NextSeq 500 as a 76 bp single end run, using one High Output v2 kit (75 cycles). All the samples were sequenced at the London Regional Genomics Center (Robarts Research Institute, London, ON, Canada) using the Illumina NextSeq 500 (Illumina Inc., San Diego, CA, USA). The Fastq data files were analyzed using Partek Flow (St. Louis, MO, USA). After importation, the data were aligned to the Homo sapiens hg19 genome using STAR 2.7.3a and annotated using Ensemble v100 after filtering for PCR duplicates. Features with less than 9 reads were filtered out, followed by normalization by CPM (Counts Per Million and add 0.0001). The fold change and *p*-values were determined using Partek Flow’s Gene Specific Analysis (GSA). The filtered lists of genes changing ≥ 1.5 fold with a *p*-value of less than 0.05 were then analyzed for enriched Gene Ontology (GO) pathway terms.

### 2.13. Statistical Analysis

Graphpad Prism v9.3.1 (Boston, MA, USA) was used for all the statistical analyses. All the in vitro experiments involved at least three biological replicates. For the antiviral assays, the determination of the 50% inhibitory concentration (IC**_50_**) was calculated using nonlinear regression (log[inhibitor] vs. response (three parameters)). For the cytotoxicity assays, the determination of the 50% cytotoxic concentration (CC_50_) was calculated u nonlinear regression (Sigmoidal, 4PL, X is concentration). *p* values less than 0.05 were deemed to be significant.

## 3. Results

### 3.1. BOLD-100 Inhibits SARS-CoV-2-Induced Cytopathic Effects

**Figure 1:**
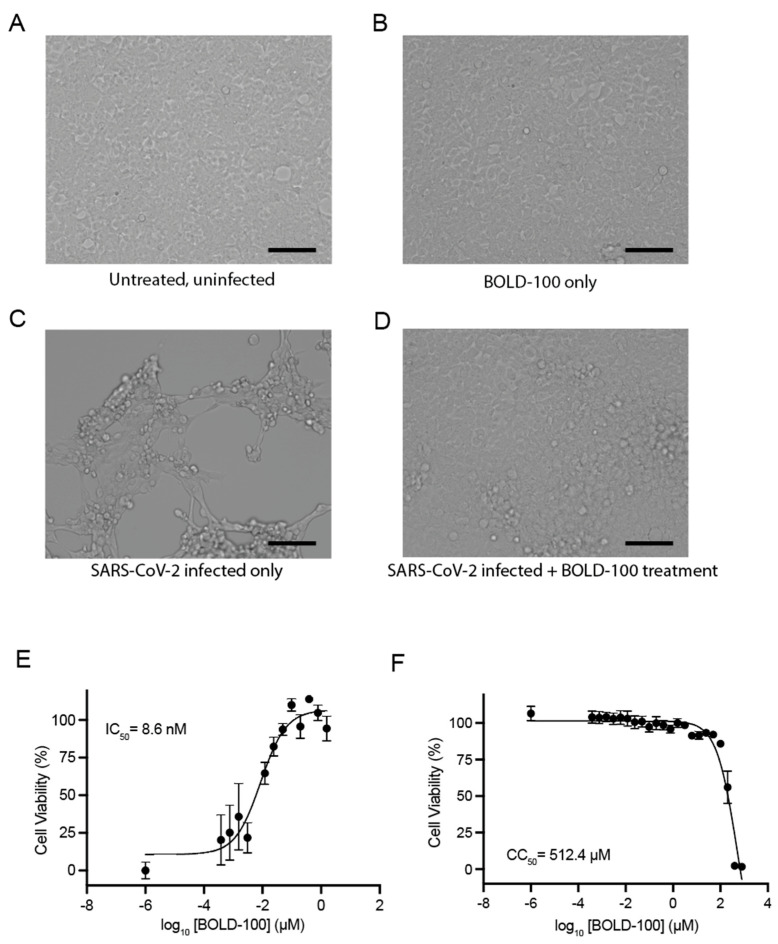
BOLD-100 inhibits SARS-CoV-2-induced cytopathic effects. Vero E6 cells were infected with SARS-CoV-2 (2019-nCoV/USA-WA-1/2020; “Wuhan isolate”) at MOI = 0.01 and treated with varying concentrations of BOLD-100. (**A**–**D**) Representative brightfield images (20× magnification, EVOS M7000 imaging system) of Vero E6 cells at 48 h post-infection: (**A**) uninfected and untreated control cells, (**B**) uninfected cells treated with 100 µM BOLD-100, (**C**) untreated cells infected with SARS-CoV-2, and (**D**) cells infected with SARS-CoV-2 and treated with 100 µM BOLD-100. Scale bars represent 100 μM. (**E**) Relative cell viability levels were measured at 72 h post-infection using the CellTiter-Glo cell viability assay (Promega). Results are presented as mean ± SEM of 4 independent experiments. RLU, relative light units. (**F**) Relative cell viability of uninfected cells treated with varying concentrations of BOLD-100 at 72 h post-treatment. Results are presented as mean ± SEM of 4 independent experiments. The IC_50_ and CC_50_ values were determined by nonlinear regression.

As an initial measure of antiviral activity, we evaluated the ability of BOLD-100 to inhibit the cell death induced by SARS-CoV-2. The Vero E6 cells, which are highly susceptible to SARS-CoV-2-induced cytopathic effects, were infected with SARS-CoV-2 at a multiplicity of infection (MOI) of 0.01, an MOI that, in our experiments, induced near-complete cytopathogenicity in this cell line at 72 h post-infection. The infected cells were treated with varying concentrations of BOLD-100. At 72 h post-infection, the cell viability was examined relative to the mock-treated cells. Representative brightfield microscopy images demonstrate the characteristic cytopathic effect caused by SARS-CoV-2 infection in the Vero E6 cells and the protection afforded by BOLD-100 (Figure 1A–D). To quantitatively assess the impact of BOLD-100 on cell survival following a SARS-CoV-2 infection, we repeated this experiment and measured the ATP levels in the infected cells using a CellTiter-Glo assay (Promega) as a surrogate measurement of viability. In this assay, the presence of ATP leads to the production of luminescence by an engineered luciferase. The amount of relative light units (RLUs) produced in a well is proportional to the number of viable cells. As shown in Figure 1E, BOLD-100 exhibited a dose-dependent inhibition of SARS-CoV-2-induced cytopathic effects (IC_50_ = 8.6 nM).

In parallel, we used the same assay to measure the cytotoxicity of BOLD-100 in the absence of infection. At 72 h post-treatment, the cells treated with ≤100 µM of BOLD-100 remained viable at similar levels to the mock-treated cells. At 200 µM of BOLD-100, approximately 51.9% of the cells remained viable after 72 h, while at ≥400 µM of BOLD-100, no viable cells were detected (Figure 1F). Thus, the inhibitory effects of BOLD-100 against SARS-CoV-2 occur well below the drug’s toxic range. Taken together, these data show that BOLD-100 treatment protected the cells from SARS-CoV-2 cytopathogenicity at nanomolar concentrations, indicating antiviral activity in vitro.

### 3.2. BOLD-100 Exhibits Dose-Dependent Inhibition of SARS-CoV-2 Replication

**Figure 2:**
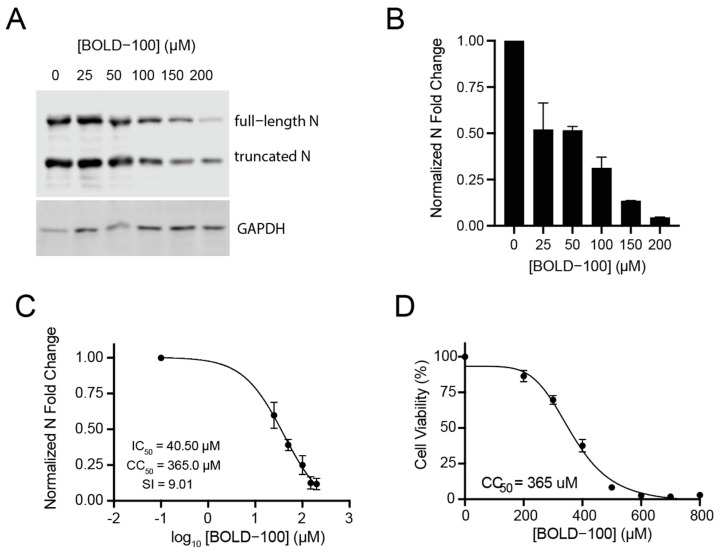
BOLD-100 inhibits intracellular accumulation of SARS-CoV-2 N protein and RNA. Human 293T-ACE2 cells were infected with SARS-CoV-2 (2019-nCoV/USA-WA-1/2020; “Wuhan isolate”) (MOI = 0.001) and treated with varying concentrations of BOLD-100. (**A**) After 48 h of infection, expression of SARS-CoV-2 N protein was analyzed by separating cell lysates on SDS-PAGE gels followed by immunoblotting with anti-N antibodies. See Figure S1 for uncropped image. (**B**) Band densities were quantified using ImageJ (NIH) and normalized to GAPDH as a loading control. Bars represent the mean ± SD of the normalized fold changes over mock treated cells. Representative Western blot of two independent experiments shown. (**C**) Relative levels of SARS-CoV-2 N RNA were analyzed using quantitative RT-PCR and normalized to GAPDH as a loading control. Results are presented as mean ± SEM of 3 independent experiments. (**D**) Relative cell viability was determined using the CCK8 assay (Sigma). Data represent mean ± SEM of 4 independent experiments. The IC_50_ and CC_50_ values were determined by nonlinear regression.

Given that BOLD-100 inhibited SARS-CoV-2-induced cytopathic effects, we next asked whether BOLD-100 treatment would inhibit SARS-CoV-2 replication in cell culture. While Vero E6 is a practical cell line for propagation and antiviral assays with SARS-CoV-2, it is not human in origin. To strengthen the relevance of our data to future clinical applications, we chose to use human cells for the subsequent experiments. We obtained human embryonic kidney 293T (HEK 293T) cells stably transduced with a lentiviral vector encoding ACE2, the receptor for SARS-CoV-2 entry (293T-ACE2 cells). SARS-CoV-2 naturally exhibits renal tropism, justifying the use of these 293T-ACE2 cells as an infection model [41,42,43]. The human 293T-ACE2 cells were infected with SARS-CoV-2 at an MOI of 0.001 for 1 h and treated with varying concentrations of BOLD-100. At 48 h post-infection, the cell lysates were harvested and the levels of SARS-CoV-2 nucleocapsid (N) protein and RNA at each BOLD-100 concentration were measured to determine the effect of BOLD-100 on SARS-CoV-2 replication. A quantitative Western blot analysis using a monoclonal antibody directed towards SARS-CoV-2 N protein demonstrated that BOLD-100 treatment inhibited the intracellular accumulation of the N protein in a dose-dependent manner (Figure 2A,B). N protein is highly abundant and is a critical factor for the production of infectious virions, owing to its role in packaging the viral genome [44,45]. Likewise, RT-qPCRs indicated a dose-dependent reduction in N RNA following BOLD-100 treatment (IC_50_ = 40.5 µM) (Figure 2C). At the highest BOLD-100 concentration tested (200 µM), this effect corresponded to a 21.9-fold decrease in N protein and an 8.5-fold decrease in N RNA over the respective mock-treated controls (Figure 2A–C).

To measure the BOLD-100 cytotoxicity in these cells, we treated them with cell culture media containing varying concentrations of BOLD-100 or cell culture media with 0.1% DMSO as a mock-treated control. Forty-eight hours post-treatment, we measured the relative cell viability at each BOLD-100 concentration via the CCK8 assay. This analysis revealed that the 50% cytotoxic concentration (CC_50_) of the BOLD-100 in the 293T-ACE2 cells was 365 µM (Figure 2D). In concordance with our cytopathic protection assays, these inhibitory effects occurred at relatively non-toxic concentrations of BOLD-100. The selectivity index (SI) (IC_50_/CC_50_), a measure of a drug’s antiviral activity compared to its cellular toxicity, was calculated to be 9.0. Taken together, these data show that BOLD-100 inhibits the production of SARS-CoV-2 particles.

### 3.3. BOLD-100 Inhibits SARS-CoV-2 Variants of Concern

Next, we tested if BOLD-100 exhibited antiviral activity towards three SARS-CoV-2 variants of concern, alpha, beta, and delta. We infected the 293T-ACE2 cells with either the original SARS-CoV-2 Wuhan isolate, alpha, beta, or delta variants, and treated them with BOLD-100. We harvested the cell lysates at 48 h post-infection and measured the SARS-CoV-2 N RNA levels via an RT-qPCR. As shown in Table 1, BOLD-100 inhibited the alpha variant at slightly lower concentrations than the ancestral strain (IC_50_ = 35.9 µM, SI = 10.2), while the delta variant was inhibited at concentrations slightly higher than the ancestral strain (IC_50_ = 46.9 µM, SI = 7.8). The beta variant exhibited the highest IC_50_ out of the four isolates tested (IC_50_ = 78.2 µM, SI = 4.7), indicating that it exhibited the most resistance to the antiviral activity of BOLD-100 out of all the SARS-CoV-2 isolates tested (Table 1). Together, these results show that BOLD-100 differentially inhibited the accumulation of viral RNA in cells infected with SARS-CoV-2 variants.

### 3.4. BOLD-100 Counteracts SARS-CoV-2-Induced Changes in the Host Transcriptome

**Figure 3:**
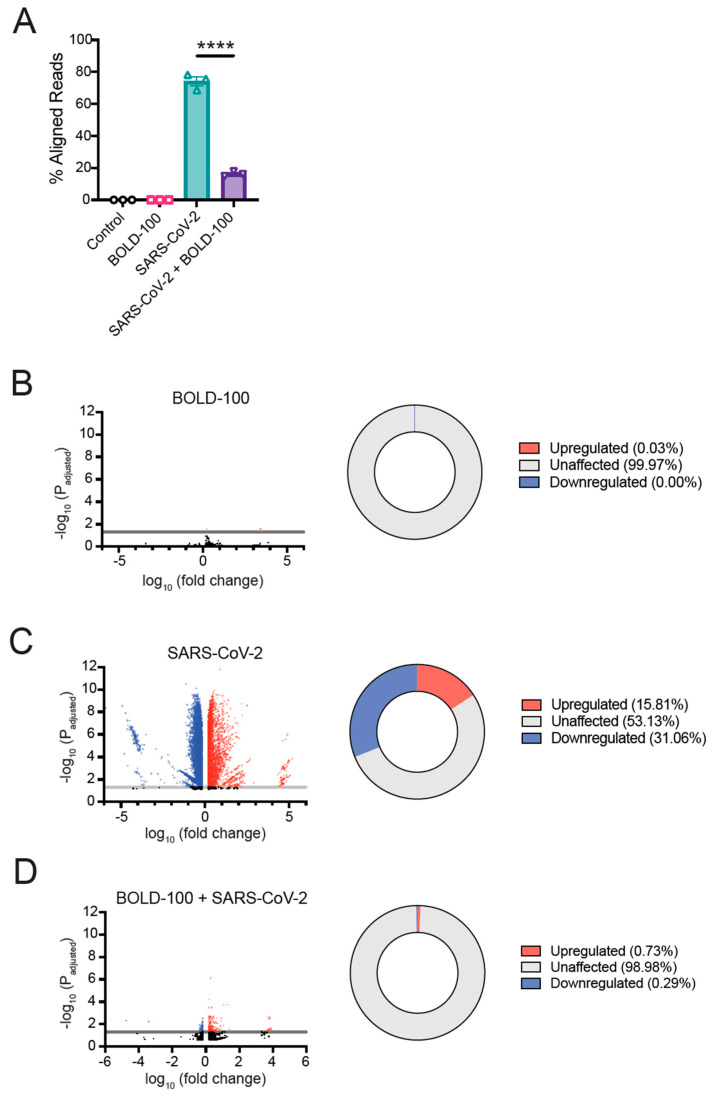
Transcriptional profile of 293T-ACE2 cells following SARS-CoV-2 infection and BOLD-100 treatment. 293T-ACE2 cells were infected with SARS-CoV-2 at MOI = 0.05 with or without 100 µM BOLD-100 for 24 h. Total RNA was extracted from cells, processed for RNA sequencing, and sequenced. Gene read counts for each treatment were normalized by the counts per million method. (**A**) Sequencing reads were aligned to either the human genome or SARS-CoV-2 genome. The percentage of virus-aligned reads over total reads was calculated and is shown as mean ± SEM. Data represent the average of 3 independent experiments. ****, *p* < 0.0001 (unpaired *t*-test). (**B**–**D**) Volcano plots showing differentially expressed genes are shown for: (**B**) cells treated with 100 µM BOLD-100 only compared to mock-treated cells, (**C**) cells infected with SARS-CoV-2 compared to mock-infected cells, and (**D**) cells treated with 100 µM BOLD-100 and infected with SARS-CoV-2 compared to SARS-CoV-2 infected cells only. Differentially expressed genes (*p*-adjusted < 0.05) with absolute fold change ≥ 1.5 are indicated in blue (downregulated genes) and red (upregulated genes). Horizontal line illustrates threshold where *p*-adjusted= 0.05. Pie charts represent proportion of upregulated and downregulated genes (*p* < 0.05) under each condition. Data represent the average of 3 independent experiments.

Next, we asked if BOLD-100 affected the cellular transcription profile of SARS-CoV-2-infected cells. The 293T-ACE2 cells were mock-infected or infected with SARS-CoV-2 (Wuhan isolate) (MOI = 0.05) in the presence or absence of BOLD-100 (100 µM) and incubated for 24 h. The total RNA was then extracted from the cell lysates and the messenger RNA (mRNA) was analyzed using an RNA Sequencing approach. As expected, the examination of the proportion of reads aligned to the SARS-CoV-2 genome under each condition showed that the uninfected cells did not yield reads aligned to the SARS-CoV-2 genome, whereas in the SARS-CoV-2-infected cells, 74.2% of the total reads detected mapped to the SARS-CoV-2 genome (Figure 3A). Notably, the SARS-CoV-2-infected cells that were subsequently treated with BOLD-100 exhibited a significant decrease in the percentage of viral reads detected (16.3%) (Figure 3A). These data correlate with our previous findings that BOLD-100 inhibits the replication of SARS-CoV-2.

To determine the specific transcriptional effects of the BOLD-100 treatment and SARS-CoV-2 infections on the 293T-ACE2 cells, we performed a differential gene analysis. Here, we defined genes as differentially expressed compared to the control (mock-treated and mock-infected) cells if the magnitude of the fold change was greater than 1.5 and if the *p*-value adjusted for false discovery rate was less than 0.05. The treatment of the cells with BOLD-100 alone did not drastically alter the cellular transcriptome, with only 0.03% of the total genes being differentially expressed compared to the untreated control (Figure 3B). For the cells treated with BOLD-100 in the absence of SARS-CoV-2 infection, the only upregulated genes that met the applied cutoffs of *p*-adjusted ≥ 0.05 and a fold-change ≥ 1.5 were *RP11-433J8.1*, *ZNF439*, *KIAA1755*, and *CABP4*. The only downregulated gene was *DHRS2*. (Tables S1 and S2). In contrast, SARS-CoV-2 infection induced large changes in the cellular transcriptome, with 46.87% of the total genes detected being differentially expressed (Figure 3C). Among the genes with the highest magnitude of upregulation after infection were *ZNF334*, *FUT6*, *KLRK1*, *IRGM*, *FCAMR*, *PPP1R1B*, *GPR111*, *CABP4*, and *PIGR* (Tables S1 and S3). Conversely, among the genes most downregulated by SARS-CoV-2 infection were *PDF*, *HBQ1*, *LMP3*, and *SPINK2* (Tables S1 and S4).

In stark contrast to the cells infected with SARS-CoV-2 alone, the percentage of genes differentially expressed in the cells infected with SARS-CoV-2 and treated with BOLD-100 was reduced to 1.02% (Figure 3D). The differentially expressed genes in the cells treated with BOLD-100 and infected with SARS-CoV-2 were largely shared with those observed in the cells infected with SARS-CoV-2 alone (Tables S1, S4 and S5). While none of these shared protein-coding genes were downregulated to a large extent, several were highly upregulated, including *GOLGA6B*, *FUT6*, *KLRK1*, *FCAMR*, *CABP4*, and *LTA* (Tables S1, S5 and S6).

**Figure 4:**
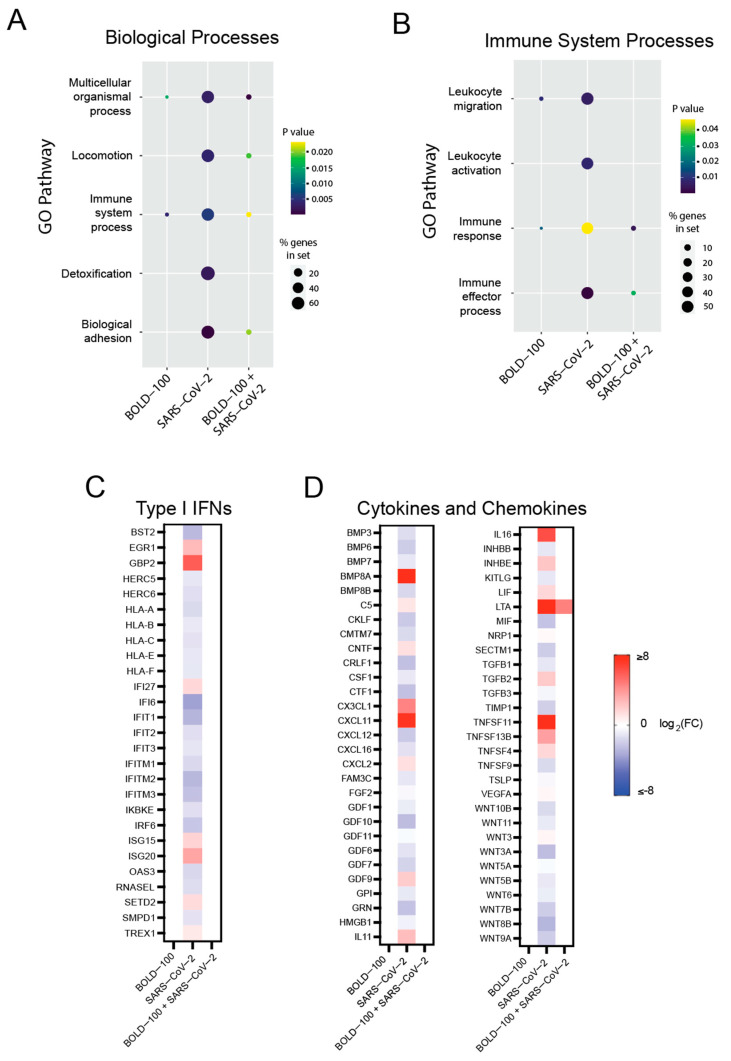
Characterization of the differentially expressed genes after SARS-CoV-2 infection and BOLD-100 treatment. (**A**,**B**) Dotplot visualization of enriched GO terms following SARS-CoV-2 infection and/or BOLD-100 treatment in 293T-ACE2 cells. 293T-ACE2 cells were infected with SARS-CoV-2 at MOI = 0.05 or mock-infected for 24 h. For cells treated with BOLD-100, 100 µM BOLD-100 was supplemented in cell culture media. Total RNA was extracted from cells, processed for RNA sequencing, and sequenced. Gene read counts for each treatment were normalized by the counts per million method. Genes with absolute fold change ≥ 1.5 and *p* < 0.05 compared to mock-treated and mock-infected cells were included. Data were analyzed for GO terms significantly enriched by SARS-CoV-2 infection at the biological processes level (GO:0008150) (**A**) and under the “immune system process” category (GO:0002376) (**B**). Data are representative of 3 independent experiments. (**C**,**D**) Heatmaps of differentially expressed genes following SARS-CoV-2 infection and/or BOLD-100 treatment in 293T-ACE2 cells. 293T-ACE2 cells were infected with SARS-CoV-2 at MOI = 0.05 or mock-infected for 24 h. For cells treated with BOLD-100, 100 µM BOLD-100 was supplemented in cell culture media. Total RNA was extracted from cells, processed for RNA sequencing, and sequenced. Gene read counts for each treatment were normalized by the counts per million method. Genes with absolute fold change ≥ 1.5 and *p* < 0.05 compared to mock-treated and mock-infected cells were included. Heatmaps depict DEGs induced by SARS-CoV-2 infection under the GO datasets for (**C**) response to type I interferon (GO:0034340) and (**D**) cytokine activity and chemokine activity (GO:0005125, GO:0008009). Legend depicts the log2-transformed fold change of DEGs, with upregulated genes shown in red and downregulated genes shown in blue. Data are representative of 3 independent experiments.

We then analyzed the enriched Gene Ontology (GO) terms under each condition to identify the key cellular pathways that were impacted by SARS-CoV-2 infection and the BOLD-100 treatment. For this analysis, we analyzed genes with a ≥ 1.5-fold change and an unadjusted *p*-value of < 0.05. Among the high-level GO terms most highly enriched by SARS-CoV-2 infection at the biological process level was “immune system process” (Figure 4A). Within this category, SARS-CoV-2 infection enriched terms related to leukocyte migration and activation, as well as the broader immune response (Figure 4B). To explore the specific impact of infection and drug treatment on these gene categories, we analyzed the expressions of the genes involved in the Type 1 IFN response, cytokine signaling, and chemotaxis. Consistent with previous reports on human primary bronchial epithelial cells, the gene enrichment analyses illustrated a generally diminished IFN-I signaling biology for SARS-CoV-2 overall, though certain IFN-induced factors were nonetheless highly upregulated (Figure 4C) [46]. Several key IFN-induced host restriction factors, including *BST2/tetherin*, *interferon-induced transmembrane proteins* (*IFITMs*), and other antiviral genes such as *IFI6* and *IFIT1,* were downregulated by SARS-CoV-2. Strikingly, these genes were not differentially expressed compared to the control in the presence of BOLD-100 (Figure 4C). Likewise, various cytokines and chemokines involved in immune response were downregulated by SARS-CoV-2 infection, including *CXCL12* and *CXCL16*, which activate and attract leukocytes, respectively, and the pro-inflammatory cytokine *MIF* (Figure 4D). However, this converse pattern was also observed in some cases, in which pro-immune cytokines and effector genes such as *CXCL11*, *IL16*, *GBP2*, *TNFSF11*, and *LTA* were strongly upregulated by SARS-CoV-2 infection. Similar to the Type I IFN-induced genes, the expressions of these cytokine and chemokine genes were at the control levels in the presence of BOLD-100, except *LTA,* whose expression remained high after the BOLD-100 treatment. Taken together, BOLD-100 treatment of SARS-CoV-2-infected cells counteracts virus-induced cellular transcriptional changes.

### 3.5. BOLD-100 Inhibits HIV-1 Replication

**Figure 5:**
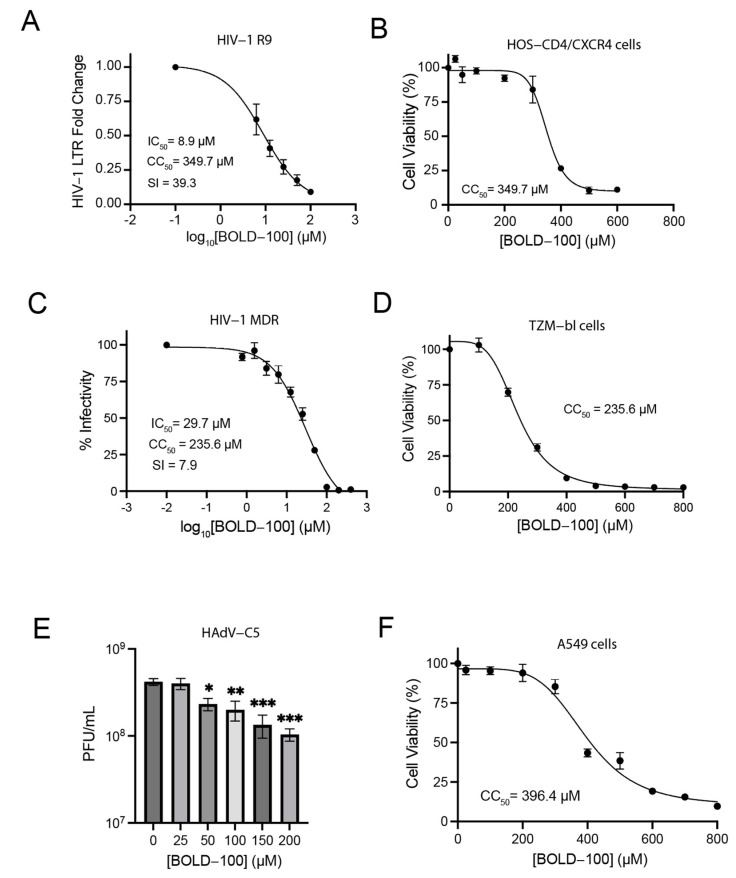
BOLD-100 inhibits replication of infectious HIV-1 and HAdV-C5. (**A**) Human HOS-CD4/CXCR4 cells were infected with replication-competent HIV-1 R9 at MOI = 0.2 for 1 h. Subsequently, cells were treated with varying concentrations of BOLD-100 and incubated for 48 h. Virus-containing supernatants were collected from infected cells and analyzed for levels of the HIV-1 (LTR) using quantitative RT-PCR. IC_50_ was calculated using nonlinear regression (log[inhibitor] vs. response, three parameters). Data are presented as mean ± SEM of fold change over diluent-treated controls and are representative of 4 independent experiments. (**B**) Uninfected HOS-CD4/CXCR4 cells were treated with varying concentrations of BOLD-100. Forty-eight hours post-treatment, relative cell viability was determined using the CCK8 assay (Sigma). Data represent mean ± SEM of 4 independent experiments. CC_50_ was calculated using nonlinear regression (Sigmoidal, 4PL, X is concentration). (**C**) TZM-bl cells were infected with HIV-1 strain AD.MDR01 (MOI = 0.05) and treated with or without varying concentrations of BOLD-100. At 48 h post-infection, β-galactosidase activity was measured in cell lysates using the Galacto-Star™ β-Galactosidase Reporter Gene Assay System (ThermoFisher). Results are presented as mean ± SEM of 4 independent experiments. IC_50_ was calculated using nonlinear regression (log[inhibitor] vs. response, three parameters). (**D**) Uninfected TZM-bl cells were treated with varying concentrations of BOLD-100. Forty-eight hours post-treatment, relative cell viability was determined using the CCK8 assay (Sigma). Data represent mean ± SEM of 4 independent experiments. CC_50_ was calculated using nonlinear regression (Sigmoidal, 4PL, X is concentration). (**E**) A549 cells were infected with HAdV-C5 (MOI = 1) for 1 h. Forty-eight hours post-infection, virus was quantified by plaque assay on A549 cells. Data are presented as mean ± SEM. One-way ANOVA followed by Holm-Šídák’s multiple comparisons tests. *, *p* < 0.05. **, *p* < 0.01. ***, *p* < 0.001; *n* = 4. Statistical differences are indicated relative to infected cells treated with diluent alone. (**F**) Uninfected A549 cells were treated with varying concentrations of BOLD-100. Forty-eight hours post-treatment, relative cell viability was determined using the CCK8 assay (Sigma). Data represent mean ± SEM of 4 independent experiments. CC_50_ was calculated using nonlinear regression (Sigmoidal, 4PL, X is concentration).

Human Immunodeficiency Virus type 1 (HIV-1) is a retrovirus that is the causative agent of the Acquired Immunodeficiency Syndrome (AIDS) epidemic. HIV-1 infects CD4+ T cells, among other immune cells, leading to the depletion of these cells and a subsequent deficiency in cellular immunity [47,48]. As an initial measure of the potential of BOLD-100 as an antiviral against a more evolutionarily diverse virus like HIV-1, we evaluated its ability to inhibit viral replication in vitro. We infected HOS-CD4/CXCR4 cells with replication-competent laboratory-adapted HIV-1 R9 for 48 h in the presence and absence of BOLD-100 treatment. HOS-CD4/CXCR4 is a human osteosarcoma cell line that expresses the HIV-1 receptor CD4 and its chemokine co-receptor CXCR4 and supports robust HIV-1 replication. Subsequently, we quantified the relative abundance of the viral particles released into the cell supernatant using an RT-qPCR. As shown in Figure 5A, BOLD-100 treatment inhibited the production of HIV-1 particles from the cells in a dose-dependent manner, with an IC_50_ value of 8.9 µM and an SI of 39.3. Concurrently, we measured the cytotoxicity of BOLD-100 treatment alone in the HOS-CD4/CXCR4 cells, which yielded a CC_50_ value of 349.7 µM (Figure 5B).

We then asked whether BOLD-100 could inhibit the replication of a clinically relevant drug-resistant HIV-1 isolate, AD.MDR01. AD.MDR01 is an HIV-1 subtype B clone associated with rapid disease progression and resistance to existing HIV therapeutics. Since cells infected with HIV-1 produce both infectious and non-infectious viral particles [49,50], we utilized the infectious HIV-1 indicator cell line TZM-bl, which is a HeLa derivative that expresses the HIV-1 receptor CD4 and CCR5 co-receptor. In addition, TZM-bl cells contain a β-galactosidase and firefly luciferase reporter cassette under the transcriptional control of an HIV-1 Tat-responsive long terminal repeat promoter (LTR). When these cells are infected with HIV-1, the incoming HIV-1 Tat protein activates expression from the LTR-containing reporter construct. This approach enables the quantification of infectivity in cell culture by measuring the β-galactosidase activity. The cells were infected in the presence of BOLD-100 for 48 h, after which, the β-galactosidase activity was measured. As shown in Figure 5C, the BOLD-100 treatment inhibited the infectious AD.MDR01 production in a dose-dependent manner, with an IC_50_ of 29.7 µM and an SI of 7.9. In parallel, we measured the cytotoxicity of BOLD-100 treatment alone in the TZM-bl cells, which yielded a CC_50_ value of 235.6 µM (Figure 5D).

### 3.6. BOLD-100 Inhibits HAdV-C5 Replication

Adenoviruses are non-enveloped viruses with double-stranded DNA genomes that infect a wide range of vertebrates. Depending on their species and type, human adenoviruses have various tropisms, where, most commonly, the respiratory tract, gut, and eye are targeted [51]. Human adenovirus serotype 5 (HAdV-C5) belongs to species C and is a highly prevalent and clinically relevant virus that is mainly associated with respiratory disease [51]. We asked whether BOLD-100 inhibits the production of infectious HAdV-C5 in cell culture. A549 human lung carcinoma cells were infected with HAdV-C5 at an MOI of 1 and treated with or without varying concentrations of BOLD-100. At 48 h post-infection, the cells were harvested and the viral titers were determined using a plaque assay. The BOLD-100 treatment yielded a dose-dependent inhibition of HAdV-C5 replication, with a statistically significant reduction in the viral load detected at concentrations of 50 µM of BOLD-100 and above (*p* < 0.001, one-way ANOVA, and Holm–Šidák’s multiple comparisons test) (Figure 5E). At 200 µM of BOLD-100, this effect corresponded to a 0.6-log10 reduction in the viral titer. In parallel, we measured the cytotoxicity of BOLD-100 treatment alone in the A549 cells, which yielded a CC_50_ value of 396.4 µM (Figure 5F).

## 4. Discussion

The COVID-19 pandemic prompted an unprecedented effort to discover and repurpose therapeutics for the treatment of COVID-19. In this study, we identified BOLD-100 as a novel antiviral agent that inhibits SARS-CoV-2 replication, in addition to virus-induced cytopathogenicity and transcriptional effects. BOLD-100 retained its antiviral efficacy against major SARS-CoV-2 variants of concern and the evolutionarily diverse viruses HIV-1 and HAdV-C5, indicating the potential for its broad-spectrum antiviral activity.

The cytopathic effects (CPE) induced by SARS-CoV-2 infection in various cell lines are mainly attributable to the apoptosis of infected cells and the formation of syncytia by the interaction of the viral spike protein with the receptors on adjacent cells [52,53]. Any compound that inhibits one or more stages of the viral life cycle, such as entry, genome replication, or egress, will affect the overall ability of the virus to replicate, infect new target cells, and induce CPE. We showed that, in addition to inhibiting CPE, BOLD-100 potently inhibited the production of SARS-CoV-2 particles at concentrations well below the drug’s cytotoxic range. BOLD-100 retained its antiviral activity against major SARS-CoV-2 variants of concern. It is currently unknown why the beta variant was less susceptible to the BOLD-100 treatment compared to the other isolates. Since it is also unknown if BOLD-100 directly interacts with viral proteins, it is difficult to predict how mutations in specific viral genes will impact the drug’s efficacy. Nevertheless, the reduced efficacy of BOLD-100 against the beta variant may have been due to the key mutations present in the beta variant that improve its replication and/or entry [54]. Indeed, the beta variant has exhibited an increased potential for entry, replication, and pathogenesis in animal models compared to earlier strains [55]. While this variant’s enhanced replicative capabilities may have contributed to the diminished effect of BOLD-100, it is unsurprising that the drug still retained a measure of efficacy against the virus. Various non-antibody-based therapeutics have consistently retained their activity against major variants of concern, though their relative efficacies have been reported to vary slightly [5,56,57]

Our RNA sequencing analysis revealed that SARS-CoV-2 induces a dramatic effect on the overall cellular transcriptional response, which was counteracted by the BOLD-100 treatment. Similar to previous studies [46,58,59], our results indicated an imbalanced host response to SARS-CoV-2 infection alone, in which certain IFN-induced and immune-related genes were upregulated by the infection, whereas other important factors were downregulated. BOLD-100 exhibited little effect on the host’s transcriptional profile in the absence of SARS-CoV-2 infection, while in the presence of infection, it drastically altered these virus-induced transcriptional effects. Based on the parameters of our analysis, only five genes were differentially expressed compared to the control after the BOLD-100 treatment in the absence of SARS-CoV-2 infection: *DHRS2*, *ZNF439*, *KIAA1755*, *CABP4*, and *RP11-433J8.1*. *DHR2* encodes a dehydrogenase that reduces dicarbonyl compounds, *ZNF439* encodes a potential transcription factor, *KIAA1755* encodes a protein of unknown function, and *CABP4* regulates the calcium and neurotransmitter levels in photoreceptor synaptic terminals [60,61]. Overall, none of these genes upregulated by the BOLD-100 treatment alone have been shown to exhibit antiviral activity.

The genes highly downregulated by SARS-CoV-2 infection in the absence of BOLD-100 are key players in innate immune response. For instance, *BST2* encodes tetherin, an IFN-induced restriction factor that inhibits the egress of enveloped viruses such as SARS-CoV-2 [62]. The expressions of the antiviral genes *IFITM1*, *IFITM2*, and *IFITM2* were also downregulated by SARS-CoV-2. IFITMs have previously been shown to restrict the syncytia induced by SARS-CoV-2 and other coronaviruses [63]. The transcriptional rescue of these genes by BOLD-100 treatment may have contributed to the inhibition of CPE. *IFIT1* encodes a protein that binds viral single-stranded RNA and recruits other IFN-induced factors, while *IFI6* encodes a factor that inhibits the entry of Hepatitis C virus [64,65]. An especially relevant gene that was highly downregulated by SARS-CoV-2 was *TLR3*. This gene encodes a pattern recognition receptor that recognizes double-stranded RNA, a key viral PAMP induced in response to SARS-CoV-2 replication, and activates the antiviral signaling pathways [66]. Overall, the propensity of BOLD-100 to inhibit SARS-CoV-2-induced transcriptional changes may, in part, have alleviated the dysregulation of the innate immune response. The mechanism by which BOLD-100 influenced the transcriptional response is unknown. Although the transcriptional signature of SARS-CoV-2 infection was largely reversed by BOLD-100 treatment, approximately 16% of the reads detected in the infected and BOLD-100-treated cells were SARS-CoV-2 in origin. It may be that the inhibition of SARS-CoV-2 replication by BOLD-100 restricted the replication to levels insufficient for inducing strong, virus-induced signaling events. Alternatively, BOLD-100 may have inhibited transcription factors that become upregulated only during situations of cellular stress, such as infection. As such, BOLD-100 treatment in the absence of infection would not induce overt transcriptional effects, as we observed. Additional research is needed to gain a better understanding of the precise steps in the viral lifecycle that are affected by BOLD-100.

It is interesting that, among the immune-related genes, the *LTA* expression remained high after infection, even in the presence of BOLD-100. This gene encodes lymphotoxin-α (also known as tumor necrosis factor-β), a secreted cytokine that mediates inflammatory and antiviral responses [67]. We observed a similar pattern with *KLRK1* and *FCAMR*, which were also induced by SARS-CoV-2 infection and remained highly expressed after BOLD-100 treatment. *KLRK1* encodes NKG2D, an activating receptor for natural killer cells and other cytotoxic cells, which recognizes “induced-self” peptides presented by virus-infected cells [68]. *FCAMR* encodes an Fc receptor that binds immunoglobulin M and immunoglobulin A and is predicted to contribute to adaptive immune responses [69,70]. Overall, though BOLD-100 generally reversed the changes in the immune-related genes induced by SARS-CoV-2, it is intriguing that certain genes important in immune response were still highly expressed, which could be beneficial in a therapeutic context. The cellular response to SARS-CoV-2 is highly dynamic over the course of an infection [58]. A limitation of our study is that we analyzed the cellular transcriptional changes at one timepoint during infection. The cellular response to SARS-CoV-2 is highly dynamic over the course of an infection [58]; therefore, we only provided an analysis of the transcriptional changes during the early course of infection and the corresponding effects of BOLD-100.

To better understand the mechanism of the anti-cancer activity of BOLD-100, Schoenhacker-Alte and colleagues (2017) screened 23 cell lines and compared the cell lines that were sensitive and low-responsive to BOLD-100 treatment after 6 h [71]. They found that the sensitive cell lines were characterized by “response to chemical stimuli,” “regulation of cell death (apoptosis-inducing)”, and “chromatin organization,” whereas the low-responsive cells preferentially activated the pathways controlling the cell cycle, DNA repair, and metabolism. Although their screen did not include HEK293T cells and we treated cells for 24 h as opposed to 6 h, the related GO pathways of the genes affected by BOLD-100 treatment only were consistent with the category of low-responsive. For example, ZNF439 is involved in DNA transcription, CABP4 is involved in calcium binding, and DHRS2 is involved in metabolic processes, responses to stress, cell differentiation, and the negative regulation of apoptosis. A recent gene expression profiling study by Baier and colleagues (2022) showed that BOLD-100 is involved in the transcriptional deregulation of carbohydrate metabolism, which plays a critical role in cancer cell resistance to BOLD-100 [35]. Notably, viruses alter the host’s cell metabolism, especially their glucose metabolism, to increase the available energy and promote their own reproduction (reviewed in [72]). It was recently shown that the inhibition of glycolysis abolishes SARS-CoV-2 replication and the cytokine response, placing glycolysis as a key upstream event during SARS-CoV-2 pathogenesis [73,74]. It will be interesting to learn if the effects of BOLD-100 on glycolysis are also key contributors to its antiviral effects.

We determined that the antiviral activity of BOLD-100 was not specific to SARS-CoV-2 and that the drug also inhibits HIV-1 and HAdV-C5. Although the mechanism of this antiviral activity in these viruses remains to be characterized, our findings highlight the potential of BOLD-100 as a broad-acting antiviral. Previous research into ruthenium drugs as antimicrobials has predominantly focused on their antibacterial or antiparasitic properties [28]. However, the COVID-19 pandemic prompted a renewed effort to repurpose diverse classes of therapeutic agents for the treatment of COVID-19. In this context, several metallodrugs, including ruthenium-based compounds, were also evaluated for their antiviral potential. For instance, the BOLD-100 precursor KP1019 (indazole *trans*-[tetrachlorobis(1*H*-indazole)ruthenate(III)]) demonstrated anti-SARS-CoV-2 activity in Calu-3 human lung cancer cells [31]. Other ruthenium drugs have shown promise in an antiviral context. Several test compounds have been shown to inhibit the replication of diverse viruses, such as HIV-1 and Polio virus [75]. An identification of the other viruses that BOLD-100 does or does not inhibit will help to elucidate the specific factors or pathways targeted by the drug mechanistically.

## 5. Conclusions

We have described the antiviral potential of the ruthenium-based small molecule BOLD-100 against SARS-CoV-2 and evolutionarily divergent viruses. We used a variety of molecular techniques to show that SARS-CoV-2 replication and its cytopathic effects were inhibited in a dose-dependent manner by BOLD-100. Moreover, we showed that BOLD-100 largely reversed the transcriptional signature of SARS-CoV-2 infection in cell culture. Finally, we determined that BOLD-100 inhibited the production of infectious HIV-1 and HAdV-C5. Further studies are required to better understand the potential of BOLD-100 as a novel antiviral therapeutic to alleviate the burden of current and future viral epidemic and/or pandemics.

## Figures and Tables

**Table 1:** IC_50_, CC_50_, and SI values of BOLD-100 against SARS-CoV-2 variants of concern.

Variant	IC_50_ (µM)	CC_50_ (µM)	SI
Original isolate (Wuhan)	40.5	365.0	9.0
Alpha	35.9	365.0	10.2
Beta	78.2	365.0	4.7
Delta	46.9	365.0	7.8

## Data Availability

All data is available upon request.

## Supplementary Materials

The following supporting information can be downloaded at: https://www.mdpi.com/article/10.3390/biom13071095/s1, Figure S1: Human 293T-ACE2 cells were infected with SARS-CoV-2 (2019-nCoV/USA-WA-1/2020; “Wuhan isolate”) (MOI = 0.001) and treated with varying concentrations of BOLD-100. After 48 hours of infection, expression of SARS-CoV-2 N protein was analyzed by separating cell lysates on SDS-PAGE gels followed by immunoblotting with anti-N antibodies. **A**, LiCOR image (**A**) red, molecular weight marker (lane 1) and GAPDH; green, SARS-CoV-2 N protein. **B** and **C**, greyscale images showing only the green channel (**B**) or red channel (**C**). Relates to Figure 1 in the main text. Table S1: List of differentially expressed genes in SARS-CoV-2 infected cells in the presence or absence of BOLD-100; Table S2: Differentially expressed genes in cells treated with BOLD-100 in the absence of SARS-CoV-2 infection with *p*-adjusted ≥ 0.05 and a fold-change ≥ 1.5; Table S3: Top 30 upregulated in SARS-CoV-2 infected cells with *p*-adjusted ≥ 0.05 and a fold-change ≥ 1.5; Table S4: Top 30 downregulated in SARS-CoV-2 infected cells with *p*-adjusted ≥ 0.05 and a fold-change ≥ 1.5; Table S5: Top 30 upregulated in SARS-CoV-2 infected cells treated with BOLD-100 with *p*-adjusted ≥ 0.05 and a fold-change ≥ 1.5; Table S6: Top 30 downregulated in SARS-CoV-2 infected cells treated with BOLD-100 with *p*-adjusted ≥ 0.05 and a fold-change ≥ 1.5.
