# Supplementary material for: The Metallodrug BOLD-100 Is a Potent Inhibitor of SARS-CoV-2 Replication and Has Broad-Acting Antiviral Activity
Source: Biomolecules. 2023 Jul 8;13(7):1095. doi: 10.3390/biom13071095 (PMC10377621; doi:10.3390/biom13071095)
Supplement: Supplementary file 1 [file biomolecules-13-01095-s001.zip › Figure S1.pdf]

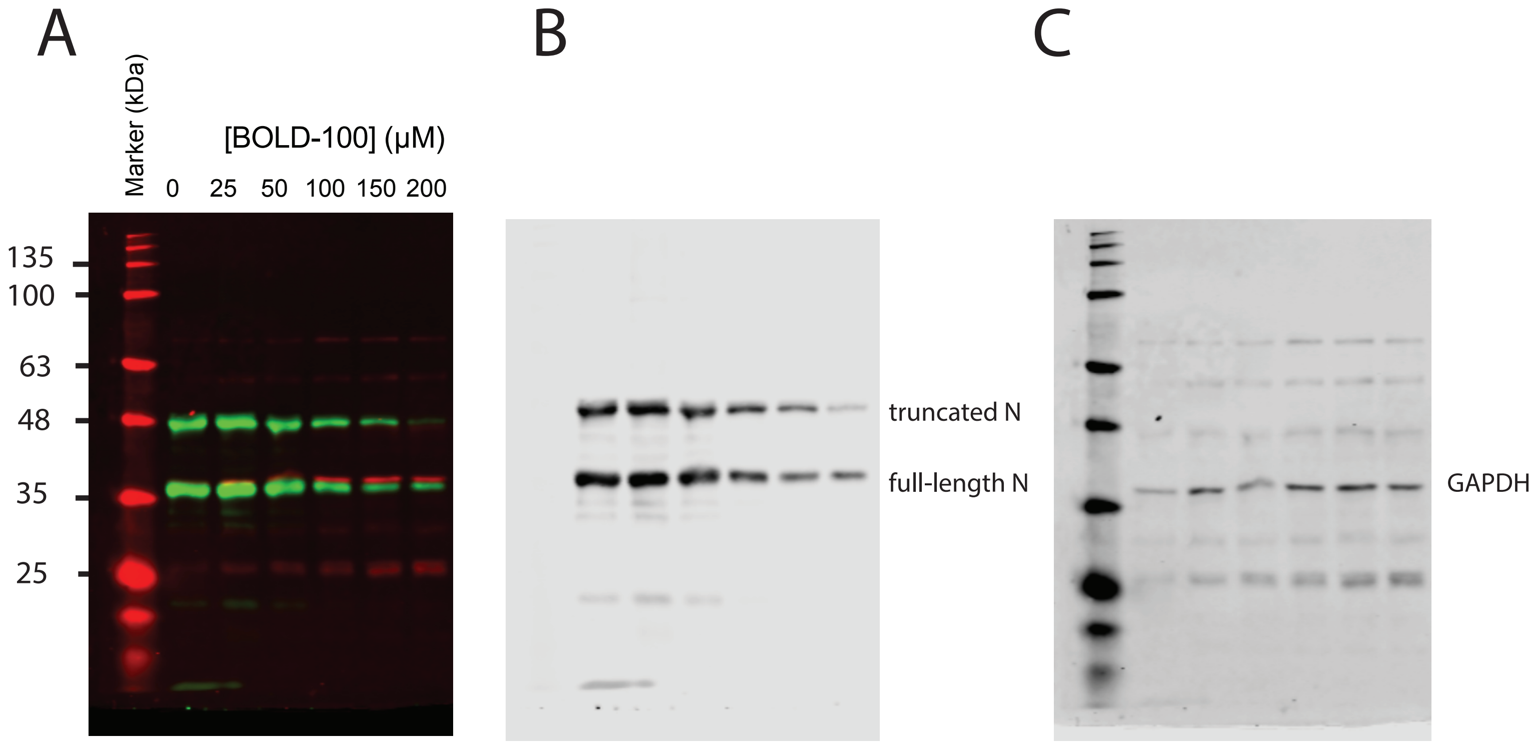

Figure S1: Human 293T-ACE2 cells were infected with SARS-CoV-2 (2019-nCoV/USA-WA-1/2020; "Wuhan isolate") (MOI=0.001) and treated with varying concentrations of BOLD-100. After 48 hours of infection, expression of SARS-CoV-2 N protein was analyzed by separating cell lysates on SDS-PAGE gels followed by immunoblotting with anti-N antibodies. A, LiCOR image (A) red, molecular weight marker (lane 1) and GAPDH; green, SARS-CoV-2 N protein. B and C, greyscale images showing only the green channel (B) or red channel (C). Relates to Figure 1 in the main text.
