# Supplementary material for: The Metallodrug BOLD-100 Is a Potent Inhibitor of SARS-CoV-2 Replication and Has Broad-Acting Antiviral Activity
Source: Biomolecules. 2023 Jul 8;13(7):1095. doi: 10.3390/biom13071095 (PMC10377621; doi:10.3390/biom13071095)
Supplement: Supplementary file 1 [file biomolecules-13-01095-s001.zip › Supplementary Tables S2-S6.pdf]

Supplementary Tables:

Table S2: Differentially expressed genes in cells treated with BOLD-100 in the absence of SARS-CoV-2 infection with P-adjusted ≥ 0.05 and a fold-change ≥ 1.5.

Rank	Gene	log_{10}(Fold Change)	$-log_{10}(P_{adjusted})$
1	RP11-433J8.1	3.57	1.37
2	ZNF439	3.50	1.37
3	KIAA1755	3.46	1.55
4	CABP4	3.39	1.55
5	DHRS2	0.23	1.55

Table S3: Top 30 upregulated in SARS-CoV-2 infected cells with P-adjusted ≥ 0.05 and a fold-change ≥ 1.5.

Rank	Gene	log_{10}(Fold Change)	$-log_{10}(P_{adjusted})$
1	VAC14-AS1	5.2	3.82
2	ZNF334	5.07	2.37
3	RP11-998D10.5	5.04	2.34
4	FUT6	5.01	3.69
5	KLRK1	4.98	3.58
6	LHFPL3-AS1	4.97	3.72
7	IRGM	4.96	2.23
8	FCAMR	4.95	3.5
9	C1orf141	4.93	2.26
10	AP001331.1	4.93	3.45
11	GOLGA6B	4.92	3.4
12	PPP1R1B	4.92	3.54
13	KIAA1755	4.91	5.9
14	GPR111	4.89	3.2
15	CABP4	4.89	6.02
16	SLC30A8	4.88	2.21
17	RP11-353N4.5	4.85	1.83

18	RP11-328C8.4	4.85	2.4
19	RP11-467L20.10	4.85	3.08
20	RP11-863K10.2	4.8	2.18
21	RP11-433J8.1	4.8	5.59
22	PIGR	4.79	2.77
23	ZG16B	4.78	2.12
24	HSH2D	4.78	3.35
25	KLRC1	4.75	2.96
26	PYY	4.73	2.09
27	AC139100.3	4.71	2.96
28	TNFSF11	4.71	3.1
29	RP11-750H9.5	4.7	1.63
30	GPR26	4.7	2.06

Table S4: Top 30 downregulated in SARS-CoV-2 infected cells with P-adjusted ≥ 0.05 and a fold-change ≥ 1.5.

Rank	Gene	$\log_{10}$(Fold Change)	$-\log_{10}(P_{adjusted})$
1	RN7SL5P	-5.89	8.37
2	PDF	-4.92	8.52
3	GS1-184P14.2	-4.8	4.28
4	RPS26P8	-4.72	7.89
5	HBQ1	-4.63	7.26
6	RP11-349N19.2	-4.61	6.46
7	RPS26P31	-4.61	3.21
8	PRDX2P4	-4.59	7.19
9	H3F3BP1	-4.59	6.84
10	RP11-680G24.4	-4.5	6.65
11	RP13-444K19.1	-4.47	7.23
12	MTX1P1	-4.46	6.4
13	IDSP1	-4.44	6.76
14	EEF1A1P4	-4.44	6.45
15	COX11P1	-4.42	6.56
16	LMO3	-4.41	6.56
17	SPINK2	-4.41	6.48

18	FTH1P20	-4.4	6.62
19	VWA5A	-4.37	6.64
20	AC005884.1	-4.37	6.55
21	SLC25A5P2	-4.34	6.58
22	HSPB1P1	-4.34	6.38
23	RP11-475I24.1	-4.34	6.38
24	CTA-963H5.5	-4.34	5.88
25	ATP5G1P4	-4.33	6.66
26	PTGES	-4.32	6.67
27	MAOB	-4.32	6.48
28	CLPSL2	-4.32	6.1
29	CACNG5	-4.31	6.2
30	TMSB4XP8	-4.29	6.32

Table S5: Top 30 upregulated in SARS-CoV-2 infected cells treated with BOLD-100 with P-adjusted ≥ 0.05 and a fold-change ≥ 1.5.

Rank	Gene	$\log_{10}$(Fold Change)	$-\log_{10}(P_{adjusted})$
1	GOLGA6B	3.93	1.47
2	LHFPL3-AS1	3.88	1.64
3	FUT6	3.87	1.56
4	KLRK1	3.84	1.54
5	KIAA1755	3.81	2.63
6	ZNF439	3.79	2.51
7	RP11-433J8.1	3.79	2.51
8	IFNA22P	3.77	1.35
9	PPP1R1B	3.76	1.53
10	FCAMR	3.76	1.49
11	GPR111	3.72	1.34
12	VAC14-AS1	3.71	1.53
13	CABP4	3.70	2.63
14	WEE2	3.66	1.42
15	RP11-498C9.16	3.65	1.38
16	AP001331.1	3.58	1.37
17	LTA	1.40	1.54

18	FAM230A	1.39	1.39
19	RP11-1220K2.2	1.28	3.76
20	FAM71F1	1.16	1.31
21	GGTLC3	1.16	1.36
22	RP11-196G18.3	1.06	1.91
23	MGAM	1.05	3.76
24	KIAA1683	0.92	2.52
25	CASS4	0.91	2.31
26	CD200R1	0.89	1.82
27	ZNF658B	0.86	1.52
28	MOBP	0.79	1.51
29	TG	0.75	1.61
30	ANKRD26P1	0.70	2.09

Table S6: Top 30 downregulated in SARS-CoV-2 infected cells treated with BOLD-100 with P-adjusted ≥ 0.05 and a fold-change ≥ 1.5.

Rank	Gene	$\log_{10}$(Fold Change)	$-\log_{10}(P_{adjusted})$
1	GS1-184P14.2	-4.74	2.31
2	RP11-426L16.9	-3.41	2.20
3	RPL41P1	-0.38	1.94
4	RAB1C	-0.37	1.64
5	GRM4	-0.36	1.48
6	RPS15AP24	-0.35	1.48
7	AC074212.5	-0.34	1.57
8	CTD-2287O16.5	-0.33	1.55
9	LUC7L2	-0.32	1.34
10	SNRPFP1	-0.30	1.74
11	PPP1R1A	-0.29	1.91
12	MT-ATP6	-0.27	1.56
13	SPOCK1	-0.27	1.34
14	MT-CO2	-0.27	1.73
15	MT-ND4	-0.26	1.56
16	PTP4A3	-0.26	1.38
17	MT-ND5	-0.26	1.82
18	HMGN1P38	-0.25	1.50

19	MT-ND4L	-0.25	1.82
20	RP13-516M14.1	-0.25	1.53
21	TENM4	-0.23	2.09
22	YBX1P10	-0.23	1.56
23	MT-ND1	-0.23	1.42
24	IGFBP5	-0.23	1.91
25	ADRA2C	-0.23	1.52
26	ACTA2	-0.22	1.69
27	MT-CO3	-0.22	1.67
28	YBX1P1	-0.22	2.08
29	HMGN2P5	-0.22	1.54
30	ATP1B2	-0.21	1.95
